# The Effects of Hydroxypropyl Methyl Cellulose and Metakaolin on the Properties of Self-Compacting Solidified Soil Based on Abandoned Slurry

**DOI:** 10.3390/ma17122960

**Published:** 2024-06-17

**Authors:** Liang Tang, Kaijian Huang, Gong Shen, Yixin Miao, Jiansheng Wu

**Affiliations:** 1 College of Civil Engineering, Nanjing Forestry University, Nanjing 210037, China; 13770663211@163.com; 2 Wuxi Communications Construction Engineering Group Co., Ltd., Wuxi 214000, China; 3 Nanjing Kegong Coal Science and Technology Research Co., Ltd., Nanjing 210037, China

**Keywords:** self-compacting solidified soil, workability, compressive strength, hydroxypropyl methylcellulose, metakaolin

## Abstract

As a new type of backfill material, Self-compacting solidified soil (SCSS) takes the abandoned slurry of cast-in-place piles after dewatering and reduction as the main raw material, which brings a problem of coordinating the working performance with the mechanical property under the condition of high mobility. In this paper, hydroxypropyl methyl cellulose (HPMC) and metakaolin were introduced as additives to solve this problem. First, the workability and mechanical properties of SCSS were regulated and optimized by means of the water seepage rate test, the flowability test, and the unconfined compressive strength test. Second, this study also used X-ray diffraction (XRD) and scanning electron microscopy (SEM) to investigate the effects of HPMC and metakaolin on the physical phase and microstructure of SCSS. In this way, the results showed that there was a significant impact on the flowability of SCSS, that is, when the dosage reached 0.3%, the water seepage rate of SCSS was reduced to less than 1%, and the compressive strength at 7 days reached its peak. At the same time, HPMC weakened the strength growth of SCSS in the age period of 7 days to 14 days. However, the addition of metakaolin promoted its compressive strength. XRD analysis showed that the additives had no significant effects on the physical phases. And, from the SEM results, it can be seen that although the water-retaining effect of HPMC makes hydration of cement more exhaustive, more ettringite (AFt) can be observed in the microstructure. In addition, it can be observed that the addition of metakaolin can generate more hydrated calcium silicate (C-S-H) due to the strong surface energy possessed by metakaolin. As a result of the above factors, SCSS filled the voids between particles and improved the interface structure between particles, thus enhanced the compressive strength.

## 1. Introduction

During the construction of highways, railways, bridges, tunnels, and other infrastructure projects, a significant volume of slurry is utilized in the drilling process of cast-in-place piles. This slurry serves multiple purposes, including safeguarding equipment, stabilizing well walls, suspending drilling cuttings, and facilitating their removal from the hole [1]. However, the properties of the slurry gradually deteriorate over time, and once it ceases to meet the required standards, it is discarded. Consequently, a substantial quantity of discarded slurry inevitably accumulates during engineering construction [2]. These discarded muds contain substantial quantities of sand, crushed stone minerals, and various chemical additives, exhibiting a moisture content ranging from 30 to 300% [3]. Characterized by high viscosity, high moisture content, strong fluidity, and low strength, this mixture poses significant challenges for disposal. Traditionally, these muds have been dumped in large-scale landfills, leading to prolonged processing cycles, elevated costs, and potential soil erosion and damage [4,5,6,7]. When soil conditions are characterized by physical factors such as hardness and dryness, chemical factors including hypoxia and salinity, as well as biological factors like initial infection by disease organisms, plant growth is threatened. However, these marginal lands tend to nurture unique species, and research results from reference [8] indicate that species obtained from marginal lands have high potential as raw materials for the production of solid biofuels. Furthermore, the poor permeability of these engineering sludges renders them difficult to dewater and solidify naturally [9]. Consequently, the adoption of appropriate treatment methods is crucial not only for environmental protection but also for fostering the healthy and sustainable growth of the engineering industry [10,11].

Previous studies have primarily focused on recycling systems, classification, and disposal strategies for abandoned mud [12,13,14,15]. However, there is a paucity of research exploring the transformation of abandoned mud into novel materials. Currently, the utilization of mud discarded from bored piles primarily involves its conversion into cement-based construction materials [16,17,18,19,20,21], volcanic ash-based materials [22,23,24], fine aggregates [25,26,27], and brick materials [28,29]. Luis et al. [22] investigated the potential of water treatment sludge (WTS) for the development of supplementary cementitious materials (SCM). The results confirmed that WTS calcined at 600 °C possesses significant potential for producing SCM. Qian et al. [27] examined the feasibility of partially replacing sand with excess excavated soil to prepare controlled low-strength materials (CLSM). Their findings revealed that an increase in soil content leads to a reduction in the strength, fluidity, water stability, and frost resistance of CLSM. Guo et al. [28] utilized blast furnace slag to stabilize excavated soft soil for use in reclamation projects. Their results showed that the combination of blast furnace slag and lime effectively inhibits sample expansion and enhances the consolidation coefficient. Nevertheless, these resource utilization methods often encounter challenges such as high energy consumption and costs. Self-compacting solidified soil (SCSS) represents a novel type of engineering backfill material that is environmentally adaptive and leverages waste materials. Its advantages, including pumpability, high fluidity, and adjustable strength, address several limitations associated with traditional backfilling methods. Given that the slurry discarded from cast-in-place piles contains a substantial amount of water, converting it into SCSS through coagulation and dewatering treatment could effectively address the challenges related to its disposal and reuse. This approach offers notable economic and ecological benefits, making it a promising direction for future research and implementation.

Traditional solidified soil faces challenges due to high water content and poor cohesion, leading to poor flowability and excessive water permeability. For self-compacting solidified soil (SCSS) to have good self-flowing filling performance, its flowability should exceed 200 mm. Water permeability should not exceed 2% to prevent post-construction settlement. Research suggests that additives like hydroxypropyl methyl cellulose (HPMC), thermal mixed rubber, and polypropylene thickener can enhance water retention and thickening in cement-based materials, potentially improving SCSS performance [30,31,32]. Cellulose ether, specifically hydroxypropyl methyl cellulose (HPMC), effectively enhances both water retention and flowability in cement-based materials [32,33]. As the most widely used cellulose ether [34]. HPMC’s robust water retention capabilities facilitate the hydration of cement, leading to significant improvements in the mechanical strength and adhesive properties of the slurry [35,36]. Patural [33] conducted a study on the rheological and water retention properties of mortar influenced by cellulose. At a dosage of 0.27%, cellulose exhibited remarkable thickening and water retention capabilities, with the water retention capacity increasing proportionally with molecular weight. Yang et al. [37] examined the impact of HPMC on cement-based grout properties. Their findings revealed that HPMC enhanced the aggregation of grout particles, resulting in an increase in compressive strength from 2.58 to 3.59 MPa after 28 days. Bulichen et al. [38] investigated the water retention mechanism of methyl hydroxyethyl cellulose (MHEC) and discovered that at low dosages, MHEC retained water primarily by adsorbing water molecules. On the other hand, metakaolin, a mineral additive, has been shown to influence the flowability and mechanical properties of self-compacting solidified soil. Khan et al. [39] conducted a study to investigate the impact of metakaolin content on the flowability and mechanical properties of cement-based composites. Their findings revealed that the introduction of metakaolin significantly reduced the flowability of these composites, while also exerting a negative influence on their compressive strength. Yu et al. [40] delved into the performance of metakaolin-modified concrete. Their results indicated that, at a water-cement ratio of 0.6, an increase in metakaolin content led to a decrease in concrete flowability and a corresponding increase in compressive strength. These results provide important insights into the role of HPMC and metakaolin in modifying the performance characteristics of cement-based materials. In our current study, lime and cement were employed to solidify the dehydrated slurry, resulting in the production of self-compacting solidified soil. To further enhance the water seepage properties of this material, hydroxypropyl methylcellulose (HPMC) was introduced. We systematically investigated the effects of both HPMC and metakaolin on the flowability and mechanical properties of the self-compacting solidified soil. Additionally, we analyzed the underlying mechanisms through the examination of phase composition and microscopic morphology. Our aim is to provide valuable insights and guidance for the optimal preparation of self-compacting solidified soil. To tackle the difficulty in regulating the workability and mechanical properties of self-compacting stabilized soil, this research aims to improve its usability. Not only will this bring substantial environmental benefits, but it also holds the potential for significant economic gains.

## 2. Experiment

### 2.1. Experimental Materials

This experiment used abandoned slurry from a certain road and bridge project in Wuxi, Jiangsu Province, China which consists of water as the liquid phase with sand, clay, metakaolin, and chemical reagents as the solid phase. After coagulation and dewatering, the moisture content of the slurry decreased to 63.8%, and the pH value decreased to 7.0. 

The cement used in the experiment is P.O 42.5 ordinary Portland cement, and its performance indicators are shown in Table 1.

The chemical composition of quicklime and metakaolin with a particle size of 1250 mesh is shown in Table 2 and Table 3.

**Figure 1:**
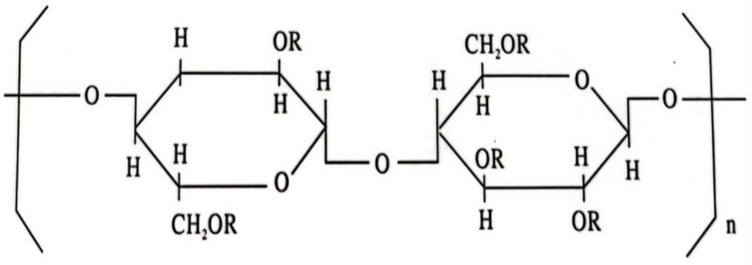
Molecular structure diagram of HPMC.

HPMC was selected with viscosities of 100,000 mPa·s and 200,000 mPa·s, denoted as HPMC–10 and HPMC–20, respectively. The molecular structure and performance parameters are shown in Figure 1 and Table 4 and Table 5.

### 2.2. Methods 

#### 2.2.1. Preparation, Curing, and Mix Design of Specimens

First, this experiment required a certain amount of abandoned slurry, which was coagulated and dewatered, and the mass of each solidifying agent according to the experimental plan needed to be calculated, weighed accurately, and mixed well. Then, the blades and mixing drum of the cement slurry mixer had to be wiped with a clean damp cloth before the experiment. Next, the abandoned slurry had to adding into the mixing drum and gradually mixed with the solidifying agent while stirring. At this step, the soil was stirred for 3 to 5 min, and casted into a 40 mm 40 mm 40 mm standard cubic mold, and the surface scrape flat with a scraper when the slurry becomes homogeneous. Lastly, the mold with the sample was put into a standard curing box for 24 h of curing, then demolded. After demolding, the sample continued to cure in the curing box for 7 days, 14 days, and 28 days. The curing box was set to standard curing conditions: temperature (20 ± 2) °C, humidity 95% [41].

In this experiment, the mix design is shown in Table 6.

#### 2.2.2. Water Bleeding Rate

The water seepage rate is a key indicator reflecting the resistance to segregation and the stability of solidified soil. The experiment was conducted with six sets of samples. When testing the water seepage rate, refer to JTG 3420-2020 “Test Code for Cement and Cement Concrete in Highway Engineering” (Chinese Standard) [42]. The solidified soil sample was placed in a beaker and allowed to stand. Every 15 min, the water seeped out from the surface of the solidified soil had to be extract by a dropper, and was measured with the mass as *m*. The calculation formula for the water seepage rate is as follows:

B=(m/M)×100%

In the formula: *B* is the water seepage rate (%) at a certain time, *m* is the mass (g) of water seepage after a certain time, *M* is the mass (g) of the original water.

#### 2.2.3. Flowability

The flowability is the main indicator for evaluating the workability of self-compacting solidified soil. In this study, the test method specified in the “Test Methods for Air-Entrained Mortar and Air-Entrained Grout” developed by the Japan Road Association was used [43]. For the HPMC experiments, six sets of samples were prepared, while for the metakaolin experiments, five sets of samples were utilized. The test apparatus was a hollow glass cylinder with an inner diameter of 80 mm and a height of 80 mm. In this experiment, the first step was placing the test cylinder on a smooth glass plate. After the self-compacting solidified soil was mixed, the mixture poured into the cylinder slowly. Then, the cylinder was measured its maximum spread diameter and vertical diameter when it was lifted slowly and evenly. The average of the two values was taken as the flowability of the solidified soil.

#### 2.2.4. Unconfined Compressive Strength

The compressive strength test method follows the standard JGJ/T 70-2009 “Standard Test Methods for Basic Performance of Building Mortars” (Chinese Standard) [44]. For the HPMC experiments, six sets of samples were prepared, while for the metakaolin experiments, five sets of samples were utilized. The test is conducted by a universal testing machine produced by Shenzhen WANCE Testing Machine Co., Ltd. (Shenzhen, China), loading at a rate of 1 mm/s until failure.

$$f_{cu}=\frac{P}{A}$$

In the formula: $f_{cu}$ is the uniaxial compressive strength of the cement soil, MPa; P is the failure load, KN; A is the cross-sectional area of the specimen, mm^2^.

Requirements for processing test results: The arithmetic mean of the measured values from three test pieces shall be taken as the average unconfined compressive strength of the test group. If the difference between the maximum or minimum measured value and the median value exceeds 15% of the median, both the maximum and minimum values should be discarded, and the median value should be taken as the compressive strength of the test group. If the difference between the two measured values and the median exceeds 15% of the median, the test results of the group are invalid, and new samples need to be prepared.

#### 2.2.5. Microscopic Analysis

The solidified soil samples were analyzed for phase composition by a combination multi-functional X-ray diffractometer (Rigaku Corporation, Tokyo, Japan). The microstructure of the solidified soil specimens was analyzed by environmental scanning electron microscopy.

## 3. Results and Discussion

### 3.1. The Effect of HPMC on the Bleeding Rate of Self-Compacting Solidified Soil

**Figure 2:**
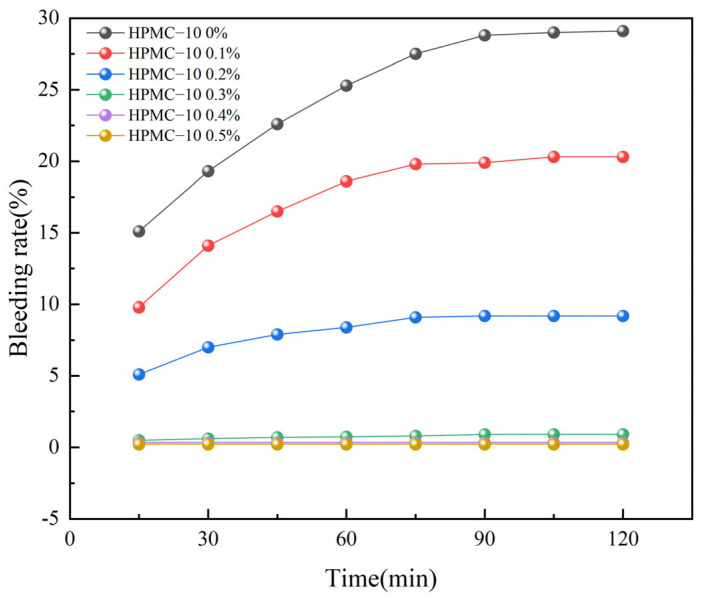
Bleeding rate of soil solidified with different dosages of HPMC–10.

**Figure 3:**
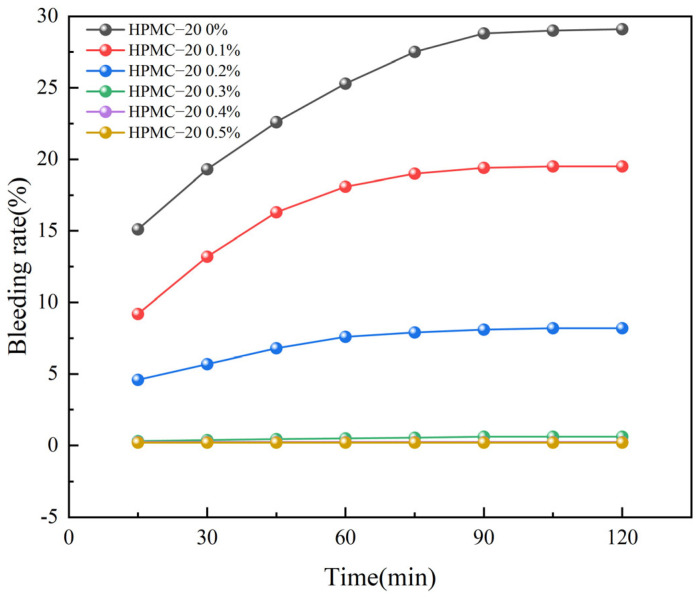
Bleeding rate of soil solidified with different dosages of HPMC–20.

From Figure 2 and Figure 3, it is evident that the blank control group, devoid of HPMC (HPMC content of 0%), presents a strikingly high water seepage rate in the self-compacting solidified soil. The water seepage rate had already escalated to 15.1% when the SCSS was mixed for 5 min. Subsequently, the seepage rate continued its upward trajectory, and ultimately stabilizing after 90 min. So, the water seepage rate of the solidified soil without HPMC peaked at 29.1 % finally, and it marked a substantial 94% surge compared to the initial rate observed at 5 min. Such a significant and sustained increase in water seepage rate is clearly unacceptable for practical engineering applications, highlighting the critical need for additives like HPMC to effectively mitigate this issue.

The water seepage of SCSS had an obvious decrease when incorporated 0.1% HPMC into the solidified soil. Specifically, the inclusion of HPMC–10 resulted in a reduced water seepage rate of 9.8% within 5 min, marking a decrease of 35.1% compared to the control group. Similarly, the addition of HPMC–20 further decreased the water seepage rate to 9.2% within the same time frame, representing a 39.1% reduction. The difference in reduction between the two types of HPMC was relatively minor. Beyond 75 min, the water seepage rates in both cases began to stabilize, ultimately settling at 20.3% for HPMC–10 and 19.5% for HPMC–20. Compared to the blank control group, this stabilization represented a 30% and 33% reduction in water seepage rate. However, it is evident that the water seepage problem remains significant, indicating the need for further optimization or the exploration of alternative solutions to achieve more satisfactory results.

As the HPMC content rose further, the water seepage rate of the solidified soil demonstrated a consistent decrease. When the HPMC content attained 0.3%, the water seepage of SCSS with two types of molecular weights of HPMC reduced to less than 1%. The water seepage rate remained stable even over time. Specifically, the water seepage rate for HPMC–10 settled at 0.92% finally, while HPMC–20 was even lower at 0.63%. Upon further increased the HPMC content SCSS to 0.5%, the final water seepage rates for it with two types of molecular weights of HPMC were exceptionally low in values at 0.21% and 0.22%, this is similar to the findings reported by Yang et al. [37]. These results clearly demonstrated that HPMC is highly effective in reducing the water seepage rate of SCSS. Additionally, the water seepage of SCSS could be decreased when using HPMC, and a slight edge in performance is evident with HPMC–20, this is in line with the results reported in reference [32].

HPMC possesses long molecular chains adorned with numerous hydroxyl and ether groups. When these chains encounter water molecules, they establish hydrogen bonds [31,45], effectively sequestering the water either internally within the molecular chains or externally on their surface, which referred as the “bound water effect” [46]. Furthermore, the ether bonds present on the molecular chains have the ability to form attachments with calcium ions situated on the surface of cement particles. This allows a single cellulose molecule to adsorb multiple cement particles, leading to a phenomenon known as the “cement particle bonding effect.” The combined influence of these two effects results in a reduction of free water within the solidified soil slurry. Consequently, the water seepage rate of the slurry is significantly decreased [47]. Based on a comprehensive analysis of test results, it has been determined that the water seepage issue in SCSS could be addressed by introduced an HPMC content of 0.3%.

### 3.2. The Influence of HPMC and Metakaolin on the Flowability of Self-Compacting Solidified Soil

#### 3.2.1. The Impact of HPMC on the Flowability of Self-Compacting Solidified Soil

**Figure 4:**
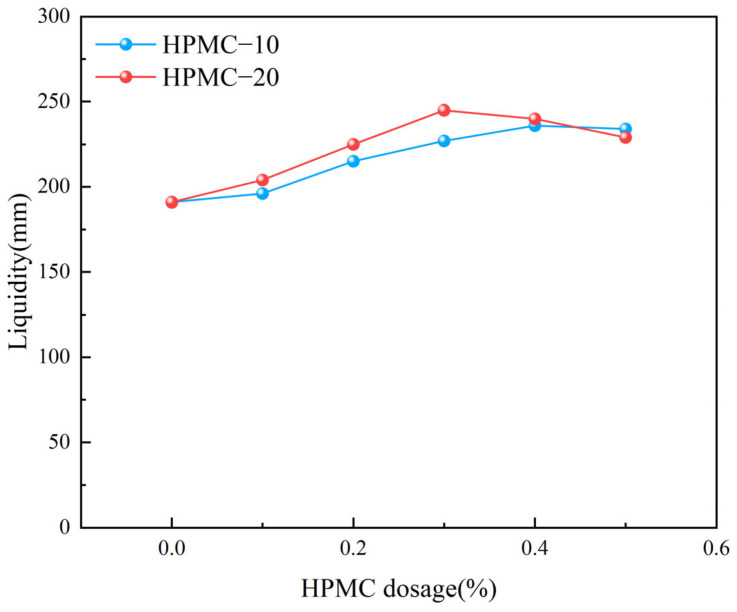
The effect of HPMC dosage on the flowability of self compacting solidified soil.

Figure 4 illustrates that as the content of HPMC with varying molecular weights rises, the flowability of self-compacting solidified soil initially improves but then gradually decreases. Notably, the influence of HPMC–20, with its higher molecular weight, is more pronounced on the flowability compared to HPMC–10. Without HPMC, the SCSS exhibited significant stratification, with a thinner slurry around the edges and cluster in the center, and resulted in poor flowability. However, the addition of 0.1% HPMC noticeably enhanced the flowability of the self-compacting soil, even it was a slow growth in flowability. 

**Figure 5:**
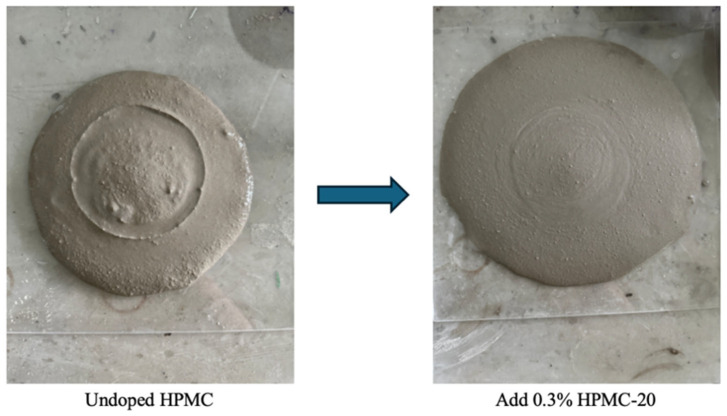
Liquidity test.

The content of HPMC increases, the flowability of the SCSS gradually improves, and the stratification phenomenon has diminished with the middle slurry collapsing towards the periphery. Figure 4 clearly demonstrates that the impact of different molecular weights of HPMC on flowability widens as the content rises. Specifically, the flowability peaks when 0.3% HPMC–20 is used, while for HPMC–10, the peak is observed at 0.4% content. Figure 5 further illustrates the significant improvement in slurry flowability, with the SCSS containing HPMC–10 achieving a flowability of 236 mm and that with HPMC–20 reaching 245 mm.

However, when the content of HPMC exceed a certain level, the flowability of the SCSS tends to decrease. This is due to the excessive viscosity caused by a high concentrations of HPMC, which in turn hinders the soil’s ability to flow smoothly [48].

#### 3.2.2. The Effect of Metakaolin on the Flowability of Self-Compacting Solidified Soil

**Figure 6:**
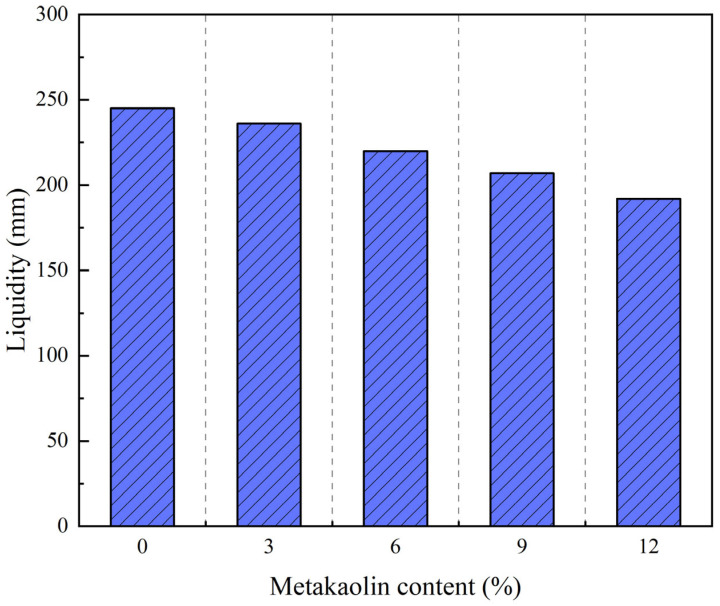
The effect of metakaolin content on the flowability of self compacting solidified soil.

Figure 6 reveals a distinct trend: as the content of metakaolin gradually increases, the flowability of the SCSS decreases, this is in agreement with the findings reported in reference [39]. Notably, as the content of metakaolin increases 3%, the flowability of SCSS has diminished by approximately 5%. When the metakaolin content reachs 12%, the flowability significantly has decreased from 245 mm to 192 mm. This observation underscores the significant impact of metakaolin on reducing the flowability of SCSS.

Decrease in the flowability of SCSS upon the addition of metakaolin is primarily attributed to two key factors. Firstly, metakaolin possesses a lamellar structure and exhibits a small particle size, enabling it to adsorb a significant amount of water. Secondly, the presence of hydrophilic minerals in metakaolin contributes to the overall viscosity of the solidified soil. The combination of these two factors enhances the viscosity of the soil, leading to a reduction in its flowability [49].

### 3.3. The Effect of HPMC and Metakaolin on the Compressive Strength of Self-Compacting Solidified Soil

#### 3.3.1. The Impact of HPMC on the Compressive Strength of Self-Compacting Solidified Soil

**Figure 7:**
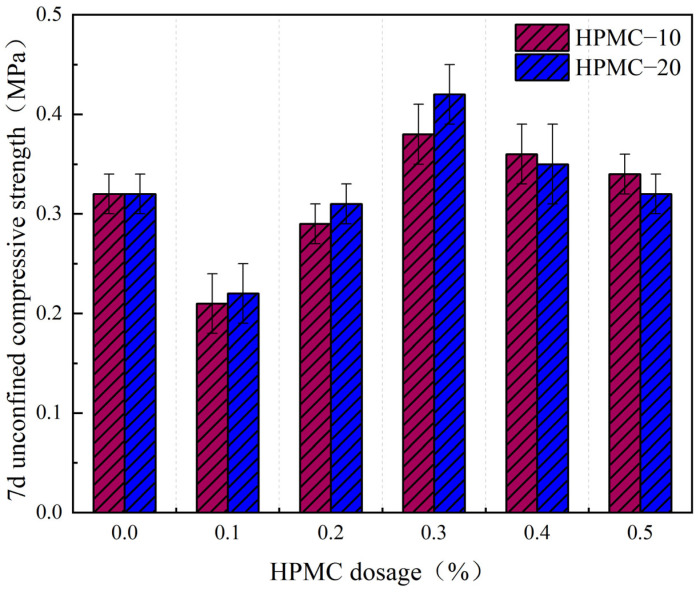
The effect of HPMC dosage on the compressive strength of solidified soil.

The thickening and water retention effects of high molecular weight thickeners significantly enhance the workability and stability of SCSS. Nevertheless, the addition of these thickeners may potentially affect the strength properties of SCSS. Figure 7 illustrates the impact of various dosages of HPMC–10 and HPMC–20 on the compressive strength of SCSS after 7 days.

From the above figure, it is observable that the compressive strength of SCSS initially decreases and subsequently increases with the augmentation of HPMC dosage, this result is consistent with the findings reported in reference [37]. Notably, the strength attains its maximum at a dosage of 0.3%. Specifically, the strength of HPMC–10 reaches 0.38 MPa, indicating a 19% elevation compared to the baseline control group (0.32 MPa). More significantly, HPMC–20 demonstrates a strength of 0.42 MPa, reflecting a substantial 31% increase over the control. This underscores the preponderance of high molecular weight HPMC in augmenting the strength of solidified soil compared to its low molecular weight variant.

However, SCSS begins to decline once the HPMC dosage surpasses 0.3%. Specifically, the strength of the SCSS with HPMC–10 diminishes by 11% compared to its peak, while the strength of the solidified soil with HPMC–20 decreases by 24% when both of them at a dosage of 0.5%. This decrement in strength can be attributed to two factors. Firstly, an excessive amount of HPMC can lead to an increase in free water within SCSS. As this water evaporates, the structural integrity of SCSS will be weakened when it leaves behind pores, which is caused by the stress concentration. Secondly, the introduction of HPMC also brings about an increase in bubbles. The viscosity of the solidified soil rises when the dosage is high, impeding the effective diffusion and elimination of these internal bubbles [50,51]. And this reduction in compactness further contributes to the decrease in strength [37]. Taken together, these factors explain the observed decline in strength when the HPMC dosage exceeds optimal levels.

**Figure 8:**
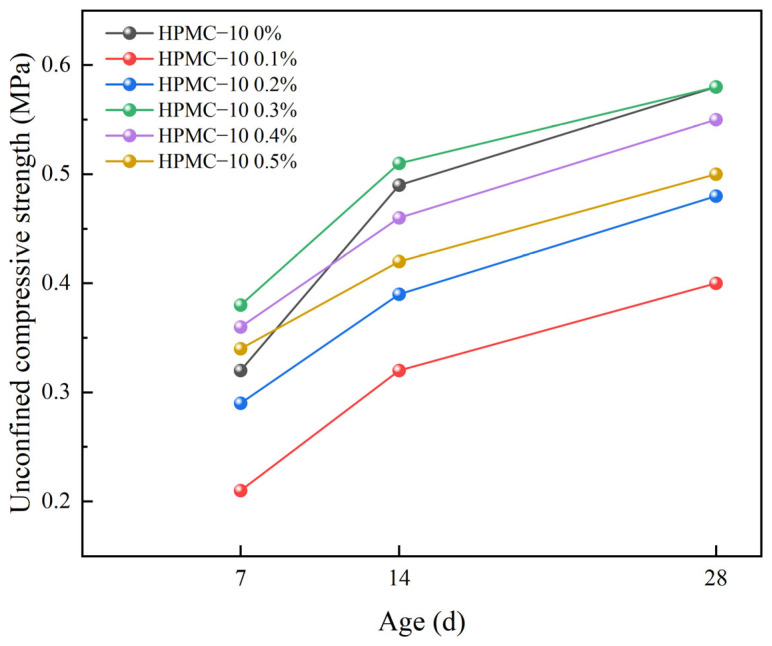
Changes in strength of solidified soil with different HPMC–10 dosages over time.

**Figure 9:**
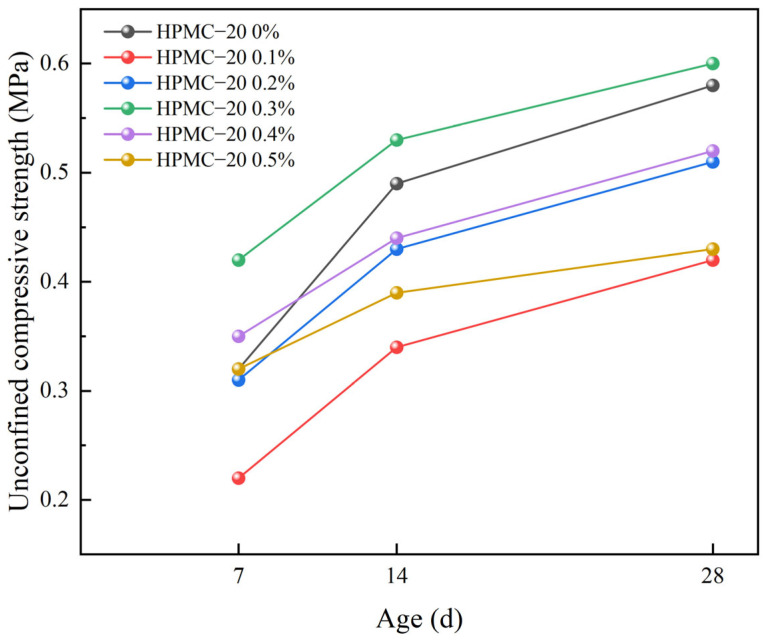
Changes in strength of solidified soil with different HPMC–20 dosages over time.

Figure 8 and Figure 9 show the variation of the strength of solidified soil with different HPMC dosages over time. The following trends can be clearly observed from the above figures: the control group had the highest strength increase from 7 to 14 days, reaching 0.17 MPa. The solidified soil with the highest HPMC dosage shows the greatest strength increase from 7 to 14 days, with a strength of 0.13 MPa for 0.3% HPMC–10, but still lower than the control group. In contrast, the strength increase is minimal for the 0.5% dosage of HPMC–20, which is 0.07 MPa during the same period, also lower than the control group, A similar phenomenon was also observed in reference [52]. Additionally, the strength increase of the control group from 14 to 28 days was 0.09 MPa, while the strength increases for different dosages of HPMC–10 and HPMC–20 solidified soil during the same period remained at around 0.08 MPa, showing a trend similar to that of the control group.

Based on this experiment, it can be deduced that the addition of HPMC hinders the strength development of self-compacting solidified soil during the initial 7 to 14 days of curing. However, from 14 to 28 days, the impact of HPMC on strength growth appears to be insignificant [50]. Furthermore, it is noteworthy that high molecular weight HPMC exerts a more pronounced influence on this trend.

#### 3.3.2. The Effect of Metakaolin on the Compressive Strength of Self-Compacting Solidified Soil

This experiment maintains a constant dosage of HPMC–20 at 0.3% while varying the metakaolin content when assessing the impact of various curing ages of metakaolin on the compressive strength of SCSS.

**Figure 10:**
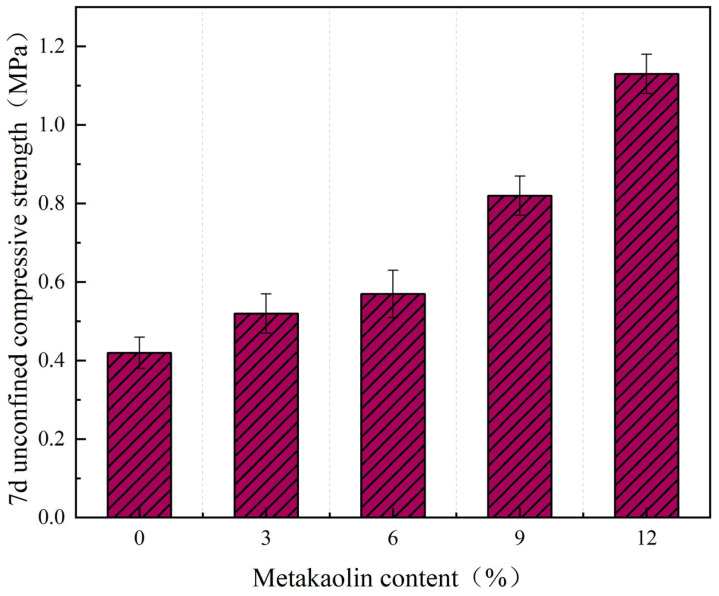
The effect of metakaolin content on the compressive strength of solidified soil.

Figure 10 illustrates the 7-day unconfined compressive strength of self-compacting solidified soil across various metakaolin dosages. Evidently, as the metakaolin dosage increases, the compressive strength of the soil also rises consistently. This result is identical to that reported in literature [40]. Notably, the compressive strength reaches 0.82 MPa when the metakaolin dosage attains 9%, surpassing the blank control group by a substantial 95%. This significant enhancement suggests that metakaolin supplementation is an effective means to boost the compressive strength of self-compacting solidified soil. And, the magnitude of this increase scales with the amount of metakaolin added.

**Figure 11:**
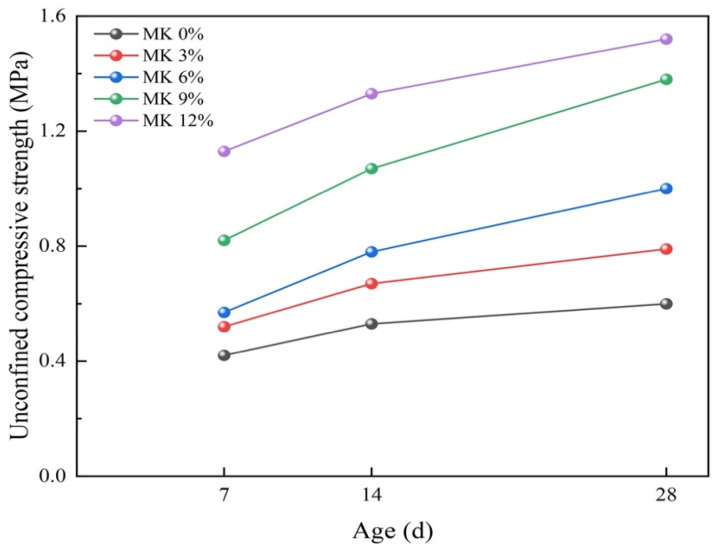
Figure of strength variation of solidified soil with age under different metakaolin contents.

Figure 11 demonstrates the compressive strength development of self-compacting solidified soil at varying ages and metakaolin dosages. Compared to the blank control group, which exhibits a strength growth of 0.11 MPa from 7 to 14 days and 0.07 MPa from 14 to 28 days, the groups with metakaolin supplementation display superior growth rates. Specifically, the metakaolin-added groups show that the lowest strength growth is 0.15 MPa from 7 to 14 days and 0.12 MPa from 14 to 28 days. The strength growth curves apparently show that the slopes of the metakaolin-containing groups are slightly steeper than those of the control, indicating that metakaolin significantly accelerates the strength growth rate of self-compacting solidified soil.

### 3.4. The Effects of HPMC and Metakaolin on the Phase Composition

**Figure 12:**
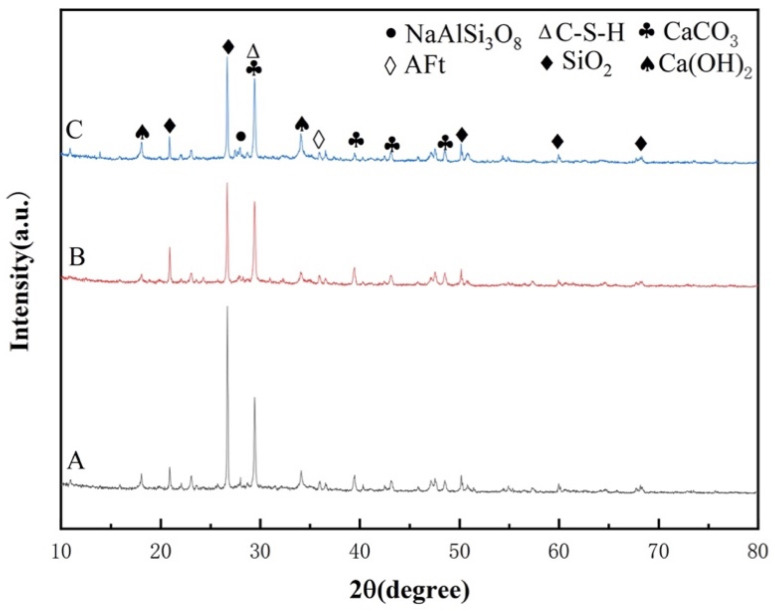
XRD spectra of solidified soil with different proportions at 7 d age.

Figure 12 presents the XRD spectra of self-compacting solidified soil with varying ratios after 7 days of curing. A, B, and C correspond to the blank group, 0.3% HPMC, and 0.3% HPMC + 3% metakaolin, respectively.

The spectra reveal the presence of SiO_2_, CaCO_3_, sodium feldspar NaAlSi_3_O_8_, Ca(OH)_2_, hydrated calcium silicate C-S-H, and ettringite AFt in all three groups. Figure 12 shows that the diffraction peaks of the SCSS remain largely unchanged across different ratios, even if the addition of HPMC and metakaolin alter the phase composition of SCSS. During the hydration process, cement produces Ca(OH)_2_ and hydrated calcium silicate C-S-H gel, while quicklime reacts with water and atmospheric carbon dioxide to form CaCO_3_. These are the primary sources of strength in the self-compacting solidified soil. And, it is obviously that the SiO_2_ peak decreases upon the addition of HPMC. This indicates that a greater quantity of active SiO_2_ is participating in the hydration reaction, leading to the formation of hydrated calcium silicate C-S-H gel and other substances [47]. Furthermore, the addition of metakaolin results in a slight strengthening of the hydrated calcium silicate C-S-H gel diffraction peak. This enhancement is attributed to the secondary hydration reaction between the active SiO_2_ in metakaolin and the hydration product Ca(OH)_2_ of cement. This reaction continuously generates hydrated calcium silicate C-S-H gel, thereby enhancing the hydration efficiency of cement.

### 3.5. The Effects of HPMC and Metakaolin on the Microstructure

**Figure 13:**
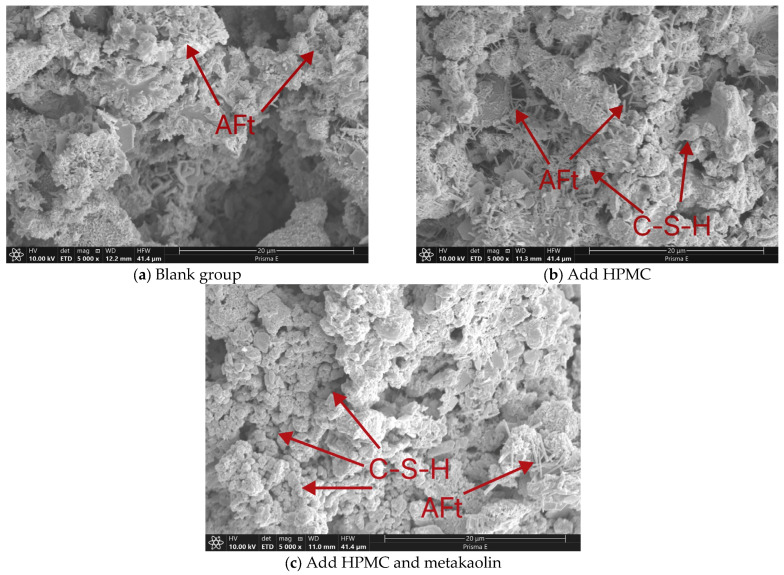
SEM images of cured soil.

Figure 13 presents the SEM image of self-compacting solidified soil with varying ratios after 7 days of curing.

From Figure 13a, it is evident that the blank control group reveals the presence of needle-like ettringite (AFt) and flocculent hydrated calcium silicate (C-S-H) gel when magnified 5000 times [53]. And, these are the primary hydration products of cement within the solidifying agent. It also shows that the gel products are interconnect and envelope the soil particles which constituting the primary source of strength for the solidified soil. Nevertheless, a notable observation from the figure is that the sample lacking additives exhibits a relatively high porosity throughout its structure.

After comparing Figure 13b, it is observed that the addition of 0.3% HPMC-20 significantly reduces the porosity in the solidified slurry sample while increasing the formation of needle-like ettringite (AFt). This is attributed to the water retention properties of HPMC, which gradually releases moisture from the slurry to the surface or air. And, the presence of free water in the solidified soil slurry extend and promote cement hydration. The expansion property of ettringite effectively fills pores, enhancing the compactness and strength of the solidified soil.

Compared with Figure 13c, SCSS shows enhanced compactness and effectively reduced porosity when it takes an addition of 3% metakaolin. The amount of flocculent hydrated calcium silicate (C-S-H) gels within the soil increases significantly. Metakaolin’s fractured chemical bonds and high surface energy promote reactions with calcium hydroxide on cement and clay particle surfaces. These reactions not only refine the particle interfaces but also incorporate additional C-S-H gels, improving the internal pore structure. Since the particles of metakaolin are smaller than the cement particles, these are able to fill in the gaps between the cement particles and the clay particles to reduce the interparticle spaces. Additionally, small amounts of needle-like AFt can be seen in filling interparticle spaces. These factors collectively contribute to a denser structure and higher compressive strength in the metakaolin-reinforced solidified soil [37].

## 4. Conclusions

This study conducted indoor experiments to investigate the effects of HPMC and metakaolin on the properties of self-compacting solidified soil. The following key findings and conclusions were obtained:

(1) Hydroxypropyl methylcellulose (HPMC) effectively reduced the bleeding rate of the soil. At an HPMC content of 0.3%, the bleeding rate was successfully brought down to less than 1%. Additionally, higher molecular weight HPMC exhibited a superior water retention capacity. Simultaneously, HPMC enhanced the fluidity of the soil, with a more significant impact observed with higher molecular weights. Specifically, peak fluidity was achieved when the content of HPMC-10 reached 0.4% and HPMC-20 reached 0.3%. However, beyond these optimal contents, the fluidity began to decline. Conversely, metakaolin had a negative impact on fluidity, with increasing metakaolin content leading to a continuous decrease in fluidity.
(2) HPMC significantly influenced the compressive strength of the solidified soil. Both molecular weights of HPMC maximized the compressive strength at a dosage of 0.3%. Exceeding this dosage resulted in a decrease in strength, with a more pronounced effect for the higher molecular weight variant. Furthermore, HPMC attenuated the strength growth of the soil between 7 and 14 days, while having minimal impact on strength growth from 14 to 28 days. On the other hand, metakaolin effectively boosted the compressive strength of the soil and facilitated strength growth throughout the entire curing period.
(3) Neither HPMC nor metakaolin had a notable effect on the phase composition of the self-compacting solidified soil. However, the water-retaining property of HPMC facilitated more thorough hydration of cement, leading to the observation of increased ettringite in the microstructure. Meanwhile, metakaolin, due to its high surface energy, reacted with calcium hydroxide on the surface of cement and clay particles, resulting in distinct microstructural changes.

## Figures and Tables

**Table 1:** Performance index of cement.

Density/ (g·cm^−3^)	Fineness (80 μm)/%	Specific Surface Area/(m^2^·kg^−1^)	Compressive Strength/MPa		Flexural Strength/MPa	
			3d	28d	3d	28d
3.11	2.6	375	23.8	48.9	5.5	9.2

**Table 2:** Chemical composition of quicklime.

Component	CaO	MgO	CO_2_	SO_2_
content/%	79	3	2	1

**Table 3:** Chemical composition of metakaolin.

Component	SiO_2_	Al_2_O_3_	Fe_2_O_3_	TiO_2_	K_2_O	MgO	CaO
content/%	52.1	44.6	0.8	1.2	0.4	0.17	0.12

**Table 4:** HPMC–10 Basic parameters.

Name	Average Molecular Weight	Viscosity/mPa·s	Ash Content	Methoxy Content	Hydroxypropyl Content
			%		
HPMC	100,000	105,100	5	22.2	10

**Table 5:** HPMC–20 Basic parameters.

Name	Average Molecular Weight	Viscosity/mPa·s	Ash Content	Methoxy Content	Hydroxypropyl Content
			%		
HPMC	200,000	192,000	5	29.5	12

**Table 6:** Mix design.

Name	Cement	Quicklime	Metakaolin	HPMC
Add quantity/%	16	15	3	0.1
			6	0.2
			9	0.3
			12	0.4
			/	0.5

## Data Availability

Data are contained within the article.
